# Potential role of heteroplasmic mitochondrial DNA mutations in modulating the subtype-specific adaptation of oral squamous cell carcinoma to cisplatin therapy

**DOI:** 10.1007/s12672-024-01445-8

**Published:** 2024-10-19

**Authors:** Amnani Aminuddin, Pei Yuen Ng, Chee Onn Leong, Suzana Makpol, Eng Wee Chua

**Affiliations:** 1 Department of Medicine, Faculty of Medicine, Universiti Kebangsaan Malaysia Medical Centre, Cheras, Kuala Lumpur, Malaysia; 2 Centre for Drug and Herbal Development, Faculty of Pharmacy, Universiti Kebangsaan Malaysia, Kuala Lumpur, Malaysia; 3 Centre for Cancer and Stem Cell Research, Institute for Research, Development and Innovation, International Medical University, Bukit Jalil, Kuala Lumpur, Malaysia; 4 AGTC Genomics, Bukit Jalil, Kuala Lumpur, Malaysia; 5 Department of Biochemistry, Faculty of Medicine, Universiti Kebangsaan Malaysia Medical Centre, Cheras, Kuala Lumpur, Malaysia

**Keywords:** Mitochondrial DNA alterations, Cisplatin resistance, Oral squamous cell carcinoma, Oxford nanopore technology

## Abstract

Cancer cells are constantly evolving to adapt to environmental changes, particularly during exposure to drug treatment. In this work, we aimed to characterize genetic and epigenetic changes in mitochondrial DNA (mtDNA) that may increase the resistance of oral squamous cell carcinoma (OSCC) to cisplatin. We first derived drug-resistant cells from two human OSCC cell lines, namely SAS and H103, by continual cisplatin treatments for about 4 months. To determine mtDNA changes induced by cisplatin, we performed nanopore sequencing and quantitative polymerase chain reaction analysis of mtDNA extracted from the cells pre- and post-treatment. We also assessed the mitochondrial functions of the cells and their capacity to generate intracellular reactive oxygen species (ROS). We found that in the cisplatin-resistant cells derived from SAS, there was a reduction in mtDNA content and significant enrichment of a m.3910G > C mutation in the *MT-ND1* gene. However, such changes were not detected in cisplatin-resistant H103 cells. The cisplatin treatment also altered methylation patterns in both SAS and H103 cells and decreased their sensitivity to ROS-induced cytotoxicity. We suggest that the sequence alterations and epigenetic changes in mtDNA and the reduction in mtDNA content could be key drivers of cisplatin resistance in OSCC. These mtDNA alterations may participate in cellular adaptation that serves as a response to adverse changes in the environment, particularly exposure to cytotoxic agents. Importantly, the observed mtDNA changes may be influenced by the distinct genetic landscapes of various cancer subtypes. Overall, this study reveals significant insights into cisplatin resistance driven by complex mtDNA dynamics, particularly in OSCC. This underscores the need for targeted therapies tailored to the genetic profiles of individual OSCC patients to improve disease prognosis.

**Supplementary Information:**

The online version contains supplementary material available at 10.1007/s12672-024-01445-8.

## Introduction

Oral squamous cell carcinoma (OSCC) is the most common subtype of oral cancer [1]. However, the management of OSCC is hampered by relapses and increased resistance of recurrent tumours to common chemotherapeutic agents, including cisplatin. Disease relapse is typically due to natural selection forces that cause tumours to be repopulated by cells that are either intrinsically refractory to therapy in a heterogenous tumour or have acquired drug resistance through survival-enhancing mechanisms [2, 3]. An important tactic adopted by cancer cells to evade drug-induced death is metabolic reprogramming, driven by a complex interplay between the mitochondrion, the nucleus, and genetic and epigenetic changes [4]. These changes, in general, underscore the functional significance of genetic heterogeneity among various subtypes of OSCC with different clinical stages and pathological types, complicating treatment and prognosis. Extensive data analysis on head and neck squamous cell carcinoma, including OSCC, revealed that high genetic heterogeneity is linked to worse clinical outcomes [5].

Mitochondria regulate a variety of cellular functions, including energy metabolism, synthesis of essential biomolecules, redox balance, reactive oxygen species (ROS) production, calcium homeostasis, stress responses, and cell death [6, 7]. Hence, mitochondrial dysfunction causes a broad range of diseases, including metabolic disorders and cancer. Accumulating evidence suggests that a major cause of mitochondrial dysfunction is abnormalities in mitochondrial DNA (mtDNA), such as point mutations, large-scale deletions, copy number variation, and methylation changes [8–10].

In this work, we performed nanopore sequencing and quantitative polymerase chain reaction (qPCR) analysis to determine the role of mtDNA alterations in the development of cisplatin resistance in OSCC, utilizing two OSCC cell lines with different clinicopathological characteristics. Changes in mitochondrial respiratory function and intracellular ROS levels were also measured, as they are key indicators of mitochondrial bioenergetics and cell survival. Elucidating the influence of cancer-specific mtDNA modifications on mitochondrial function may clarify the molecular basis of therapeutic resistance of OSCC. We anticipate that different subtypes of OSCC may exhibit distinct mtDNA profiles and varied response to cisplatin, reflecting the cell-type specific variation due to the complex genotypic and phenotypic heterogeneity among cancer cells. This valuable insight may aid in the developing better-informed treatment decisions and precise therapies targeted at the genetic profiles of individual OSCC patients for eradicating the chemotherapy failure and risk of recurrence.

## Material and methods

### Cell culture

We used two human OSCC cell lines with different clinicopathological characteristics, namely SAS (poorly differentiated, the Site, Tumour, Node, Metastasis, Pathology classification (STNMP) stage II; Japanese Cell Bank Research) and H103 (well differentiated, STNMP stage I; European Collection of Authenticated Cell Cultures). SAS cells were maintained in Dulbecco's Modified Eagle's Medium/Ham’s Nutrient Mixture F12 (DMEM/F12; Nacalai Tesque Inc., Japan) medium, supplemented with 10% fetal calf serum (GE Healthcare Life Sciences, USA) and 1% penicillin/streptomycin (Nacalai Tesque Inc., Japan) in a humidified incubator at 37 °C with 5% CO_2_. For H103 cells, 0.5 µg/mL sodium hydrocortisone succinate (Sigma-Aldrich, USA) was included in the DMEM/F12 growth medium. The absence of mycoplasma in SAS and H103 cells was confirmed using a PCR-based mycoplasma detection kit (Biological Industries Ltd., Israel) according to the manufacturer’s protocol. Short tandem repeat DNA profiling analysis was performed using the GenePrint^®^ 24 System (Promega Corporation, USA) to authenticate the cell lines used in this work.

### Cisplatin treatments

We derived cisplatin-resistant cells from the SAS and H103 cell lines via continual treatments with cisplatin (S1 Appendix), according to a published protocol [11]. The stock concentration of 2 mM of cisplatin was prepared by dissolving with 0.9% saline solution, aliquoted into microcentrifuge tubes, and stored in – 80 ℃ freezer until further use. Cells grown at a low density (approximately 20% cell confluency) were treated with 2.5 µM of cisplatin (TCI America, USA). After 24 h, the treatment was stopped, and the cells were maintained in drug-free media to allow recovery and proliferation. The cisplatin doses used were 10 and 5% of the IC_50_ previously determined in SAS and H103 cells, respectively, after 24 h of treatment with varied concentrations of cisplatin (5, 10, 20, 30, 60, and 100 µM). In the subsequent passages, the cells were re-treated using the same dose of cisplatin. The dose was doubled when the time taken for the cells to recover and become confluent was reduced by the treatment. In particular, the growth of the cell became slower after the cisplatin treatment (10–14 days to reach confluence). Gradually, the cells became confluent within 3–5 days after subsequent treatments. Overall, the cells were treated with 2.5 µM of cisplatin in the first three rounds of treatment, each lasting approximately 3 weeks. This was followed by another two rounds of treatment with 5 µM of cisplatin over 2 months. Then, the cisplatin-treated cells were maintained in drug-free media. Throughout the treatments, the cells were grown in a humidified incubator with 5% CO_2_ at 37 °C.

### Cisplatin sensitivity testing

We evaluated the drug sensitivity of the cisplatin-treated cells by assessments of cell viability, where the CellTiter 96 AQueous Non-Radioactive Cell Proliferation Assay (MTS assay; Promega Corporation, USA) was performed according to the manufacturer’s instructions. Cells were plated in a 96-well plate at a density of 5 × 10^3^ cells/well and incubated overnight at 37 °C in 5% CO_2_ humidified air. The cells were then treated with varied concentrations of cisplatin (5, 10, 20, 30, 60, and 100 µM) for 72 h [12, 13]. The absorbance was measured at a wavelength of 490 nm with Infinite 200 PRO microplate reader (Tecan Group Ltd., Switzerland). The IC_50_ of cisplatin was determined using a non-linear regression analysis of the percentages of cells that were viable after cisplatin treatment relative to that of an untreated control, against log-transformed cisplatin doses in GraphPad Prism version 7 (GraphPad Software, Inc., USA). The increase in drug resistance was confirmed by comparing the IC_50_ of the cisplatin-treated cells with that of the parental cells. The cisplatin-resistant cells derived from SAS and H103 cell lines were designated as SAS-R and H103-R, respectively, and were sometimes loosely referred to as cell lines in this article for the sake of readability.

### MtDNA sequencing

We extracted both mtDNA and nuclear DNA from 1.5 × 10^7^ cells using a QIAprep Miniprep Kit (QIAGEN, Germany). The mtDNA fraction was then enriched using solid-phase reversible immobilization paramagnetic beads (Agencourt AMPure XP; Beckman Coulter Inc., USA), according to a published protocol [14]. The concentration and purity of the mtDNA-enriched samples were measured using an OPTIZEN NanoQ Microvolume UV/Visible Spectrophotometer (Mecasys Co. Ltd, Korea). All the samples yielded A_260_/A_280_ ratios within the acceptable range of 1.7–1.9.

In total, eight MinION sequencing runs were performed for the cisplatin-resistant cells and the parental SAS and H103 cells using four MinION SpotOn Flow Cells version R9.5 (FLO-MIN107; Oxford Nanopore Technologies (ONT), UK). PCR amplicons and native DNA from the mtDNA-enriched sample were used as the sequencing templates to generate adequate depths of coverage for variant calling and to preserve nucleotide characteristics for methylation analysis. Two long PCR amplicons, ~ 8 kb in length and spanning the entire mitochondrial genome (~ 16 kb), were amplified using mtDNA-specific primers, as described in our previous work [15]. In particular, the unique mechanisms of nanopore sequencing *i.e.*, determining DNA sequences based on the disruptions in electric currents, allow for analysis of both genetic and epigenetic changes in mtDNA [16]. The details of the sequencing runs are provided in S1 Table. The DNA libraries were prepared using the 1D Ligation Sequencing Kit or 1D^2^ Ligation Sequencing Kit (SQK-LSK108 or SQK-LSK308, ONT, UK) and loaded onto the flow cells according to the manufacturer’s instructions. The duration of all sequencing runs was 48 h. The flow cells were washed using a Wash Kit (EXP-WSH002; ONT, UK) before they were reused for subsequent sequencing runs.

### Sequencing data analysis

HDF5 raw data (FAST5 format) were acquired by MinKNOW version 1.6 (ONT, UK), and local base-calling was performed using Albacore version 1.2.6 (ONT, UK), generating read files written out in the FASTQ format. Local base-calling with demultiplexing was also performed for data acquired from 1D sequencing of PCR amplicons. The quality of the base-called reads was assessed using NanoStat [17]. The base-called reads were then aligned to the human reference genome assembly GRCh38 using BWA-MEM [18] with the ont2d mode, generating alignment files (Sequence Alignment Map (SAM) format). SAMtools [19] was used to convert, sort, and index the aligned sequencing reads and convert them into BAM files, the binary version of SAM files. The mapping statistics were generated using QualiMap [20] and Geneious version 10.2.3 (Biomatters Ltd., Auckland, New Zealand) and were based on the reads aligned to the human mitochondrial genome (GRCh38) with a mapping quality score of at least 30.

Using Nanopolish [21], DNA variants were called by comparing the aligned reads with the revised Cambridge Reference Sequence of mtDNA from the human reference genome assembly GRCh38. By default setting, variants were called when the depth of variants was at least 20 and the variant frequency was at least 0.2. DNA variants with the quality-by-depth scores < 2.0 were removed. The annotation tools used to predict the functional effects of the identified variants were PolyPhen-2 [22], PANTHER [23], Envision [24], MutationAssessor [25], MutPred2 [26], and SNPs&GO [27]. The mtDNA variants identified by nanopore sequencing were cross-checked with Sanger sequencing as described in our previous study [15]. We also used Nanopolish to compute the methylation frequencies of the CpG sites within mtDNA. CpG sites with coverage of < 10 × called sites were removed. A CpG site was determined to be methylated when the log-likelihood difference, calculated by Nanopolish using a variable-order hidden Markov model, was at least 2.5. After methylated CpG sites were called, the differential CpG methylation between samples was computed using the Model-based Analysis of Bisulphite Sequencing (MOABS) [28]. A CpG site with a minimum read depth of 3 was considered differentially methylated between two samples when the credible methylation difference exceeded 0.2.

### MtDNA gene-specific qPCR

We performed qPCR to determine inter-sample differences in mtDNA content, according to a published protocol [29]. We first extracted total DNA from 5 × 10^6^ cells using the DNeasy Blood & Tissue kit (QIAGEN, Germany) according to the manufacturer’s instructions. The concentration and quality of the DNA were evaluated using the OPTIZEN NanoQ Microvolume UV/Visible Spectrophotometer (Mecasys Co. Ltd, Korea). An A_260_/A_280_ ratio of 1.7–1.9 indicated acceptable purity. Then, qPCR was performed to amplify two mitochondrial genes, tRNA^Leu(UUR)^ and 16S rRNA, and a nuclear gene, β2-microglobulin (*B2M*) using the SensiFAST SYBR No-ROX Kit (Bioline, USA). The primers used are listed in S2 Table (Integrated DNA Technologies Inc., USA). The cycling protocol comprised an initial denaturation step of 95 °C for 3 min, followed by 35 cycles of 95 °C for 5 s, 62 °C for 10 s, and 72 °C for 20 s. At the end of the PCR, an additional melting step was performed. The qPCR was performed on the CFX Connect Real-Time PCR Detection System (Bio-Rad Laboratories Inc., USA). The mtDNA content was calculated using the following equation, where ∆Cq is the difference in Cq values between mtDNA (tRNA^Leu(UUR)^ or 16S rRNA) and *B2M* genes [29].

$$MtDNA content=2 \times {2}^{-\Delta Cq}$$

### Measurement of oxygen consumption rates (OCRs)

To evaluate the difference in mitochondrial respiratory function between the cisplatin-resistant and parental cells, we measured the OCRs using the Seahorse XF Cell Mito Stress Test Kit (Agilent Technologies Inc, USA), according to the manufacturer’s instructions. Briefly, the cells were seeded on a 96-well XF cell culture microplate at a previously optimized density (SAS: 3 × 10^3^; SAS-R: 5 × 10^3^; H103 and H103-R: 3.5 × 10^3^ cells/well) and incubated overnight at 37 °C in 5% CO_2_ humidified air, to allow cell attachment. On the day of OCR measurements, the growth medium in the microplate was replaced with the culture medium provided in the assay kit, and the plate was then kept in an incubator with non-CO_2_, 37 °C humidified air for 1 h. In the meantime, the compounds required for the assay, namely oligomycin, carbonyl cyanide 4-(trifluoromethoxy) phenylhydrazone (FCCP), and rotenone/antimycin A, were prepared and loaded into a hydrated XF 96 sensor cartridge, as per the manufacturer’s instructions. The 96-well XF cell culture microplate and the XF 96 sensor cartridge were assembled and scanned using the Seahorse XF 96 Analyzer (Agilent Technologies Inc., USA). The resultant data were analyzed using Seahorse Wave Desktop Software version 2.6 (Agilent Technologies Inc., USA).

### Measurement of mitochondrial membrane potential (∆Ψm)

The evaluation of inter-sample differences in ∆Ψm required the cells to be stained with MitoTracker Red CMXRos** (**Thermo Fisher Scientific, USA). First, the cells were seeded at a density of 5 × 10^4^ cells/mL on glass coverslips in a 6-well plate and incubated overnight in 5% CO_2_ incubator at 37 °C, to allow cell attachment. The cells were then stained with 100 nM MitoTracker Red CMXRos solution for 30 min at 37 °C and fixed with 3.7% (v/v) formaldehyde (Nacalai Tesque Inc., Japan) for 15 min at room temperature. Prior to nuclear staining, the cells were permeabilised by incubation with 0.2% (v/v) Triton X-100 (Nacalai Tesque Inc., Japan) for 10 min at room temperature. To distinguish single cells, the cell nuclei were stained with 4', 6-diamidino-2-phenylindole dihydrochloride (DAPI; 1:1000; Thermo Fisher Scientific, USA) for 5 min at room temperature. Images of the stained cells were captured at 400 × magnification using the EVOS FL Auto Imaging System (Thermo Fisher Scientific, USA). The MitoTracker Red CMXRos fluorescence images were obtained using excitation and emission spectra at 585 and 628 nm, respectively. DAPI staining was visualized with an excitation wavelength of 357 nm and an emission wavelength of 447 nm. The corrected total cell fluorescence (CTCF) intensities of 30 individual cells per experimental group were measured using ImageJ version 1.50i (National Institute of Health, USA), according to the following equation [30]:

$$CTCF=Integrated\, density-(Area\times Background\, fluorescence\, mean)$$

### Measurement of intracellular ROS

We evaluated post-treatment changes in intracellular ROS levels using a fluorescence-based assay that entailed staining with a cell-permeable dye 2',7'-dichlorodihydrofluorescein diacetate (H_2_DCFDA; Sigma Aldrich Inc., USA). Briefly, the cells were seeded on a 12-well plate at a density of 5 × 10^3^ cells/well and incubated overnight at 37 °C with 5% CO_2_. The cells were treated with their respective IC_50_ doses of cisplatin for 72 h or 100 µM of hydrogen peroxide (H_2_O_2_; Bio Basic Inc., Canada) for 1 h. Then, they were harvested by trypsinization, and an equal proportion of cells in serum-free media was incubated at 37 °C with 10 µM H_2_DCFDA for 30 min for intracellular ROS staining, and MTS reagents for 4 h for cell viability analysis. Before fluorescence levels were measured, excess H_2_DCFDA stain was removed by centrifugation, and the cells were re-suspended in serum-free media, loaded into a black-96-well plate, and placed into a Varioskan Flash fluorescence microplate reader (Thermo Scientific Inc., USA). Fluorescence was subsequently captured at excitation and emission wavelengths of 504 and 529 nm, respectively. The intracellular ROS levels in the cisplatin- or H_2_O_2_-treated cells relative to an untreated control were calculated according to the following formula, which compares fluorescence intensities (F) normalised to the absorbance (Abs) values obtained from the cell viability analysis [31].

$$Relative\, fluorescence\, intensity=\frac{({F}_{Treated\, group}-{F}_{Blank\, control})/({Abs}_{Treated\, group}-{Abs}_{Blank\, control})}{({F}_{Untreated\, group}-{F}_{Blank\, control})/({Abs}_{Untreated\, group}-{Abs}_{Blank\, control}).}$$

### Statistical analyses

Data are presented as means and standard deviations of three independent experiments unless stated otherwise. The Student’s t-test was performed to compare the IC_50_ values between the cisplatin-resistant and parental cells in cisplatin sensitivity testing. The Fisher’s exact test with Bonferonni correction was performed to compare the differences in the variant allele fractions between the cisplatin-resistant and parental cells. The one-way ANOVA test with Tukey’s multiple comparisons was performed to compare the differences between the cisplatin-resistant and parental cells in mtDNA content, mitochondrial OCR, ∆Ψm and intracellular ROS. The statistical analyses were performed using GraphPad Prism version 7 (GraphPad Software, Inc., USA). Intergroup differences were considered statistically significant when *p* < 0.05 unless stated otherwise. The differences in mtDNA methylation status between the cisplatin-resistant and parental cells were assessed based on the ‘credible methylation difference’ computed by MOABS. The MOABS algorithm outperforms Fisher’s exact test, as it takes into account the sequencing depth and sample reproducibility.

## Results

### Cisplatin-treated OSCC cells showed increased level of drug resistance

We used SAS-R and H103-R cells, whose sensitivity to cisplatin was reduced by repeated drug treatments, to investigate the potential roles of mtDNA alterations in cisplatin responsiveness in OSCC. The increases in the fold resistance of SAS-R and H103-R cells relative to the parental cells were 2.02 and 1.50, respectively (Table 1).

**Table 1:** The sensitivity of SAS, H103 and their respective resistant cells towards cisplatin

OSCC cell lines	IC_50_ (µM) (Fold resistance)	
	Parental	Cisplatin-resistant
SAS	3.74 ± 0.30	7.55 ± 1.21 (2.02)**
H103	20.07 ± 1.92	30.07 ± 3.62 (1.50)**

IC_50_ is defined as the concentration of cisplatin required to reduce cell viability by half after 72 h. Higher IC_50_ values indicated lower sensitivity of the cells towards cisplatin and presumably enhanced cisplatin resistance. The fold resistance was calculated by dividing the IC_50_ of the resistant cells to that of the parental cells. IC_50_ values are presented as mean ± SD, *n* = 3. ** *p* < 0.01, when compared with their respective parental cells

### Base quality, mapping quality and depth of coverage of MinION sequencing data

In this work, eight sequencing runs were performed, where PCR amplicons and mtDNA-enriched, native DNA were analysed. The read and mapping statistics for the sequencing of SAS-R and H103-R cells are provided in Table 2. The sequencing data for SAS and H103 cells have been described previously [15, 32]. The total sequencing output varied considerably between runs, probably because of differences in the quality and performance of individual flow cells. 1D^2^ sequencing, where the same fragment is read twice, resulted in better-quality reads than 1D sequencing (Table 3). The proportion of the base-called reads with quality scores ≥ 5 was lowest (> 50%) for amplicon sequencing of H103-R cells and exceeded 99% for the other runs (Table 3). For both SAS-R and H103-R cells, sequencing of PCR amplicons and native DNA yielded adequate depth of coverage for the analysis of mutations and methylation status (Table 2; S2 Appendix).

**Table 2:** The read and mapping statistics of MinION sequencing data of SAS-R and H103-R cells

	SAS-R (PCR amplicons)	SAS-R (Native)	H103-R (PCR amplicons)	H103-R (Native)
Read statistics				
Total reads	26 132	208 721	98 377	379 325
Proportion of passed reads (%)	99.12 (25 902/26 132)	99.8 (208 315/208 721)	98.12 (96 528/98 377)	99.74 (378 337/379 325)
Total length (base)	111 818 028	870 759 097	443 430 698	1 649 487 238
Maximum length (base)	717 561	804 406	732 515	565 932
Median read length	2488	2327	2683.5	2903
Mean read length	4317	4180	4593.8	4359.8
Mapping Statistics				
Total number of mapped bases	68 139 274	746 808 302	258 249 905	1 437 593 302
Total number of bases mapped to mitochondrial chromosome	50 165 461	12 282 182	163 570 485	3 645 175
Proportion of bases aligned to mitochondrial chromosome (%)	73.62 (50 165 461/68 139 271)	1.65 (12 282 182/746 808 302)	63.34 (163 570 485/258 249 905)	0.25 (3 645 175/1 437 593 302)
Average depth of coverage	2145.4	377.2	6130.3	155.3
Pairwise identity (%)	60.1	63.4	63.2	66.1

**Table 3:** The number and fraction of reads with a quality score > 5 or > 10, as assessed via NanoStat

Sequencing chemistry	1D sequencing chemistry				1D^2^ sequencing chemistry			
Flow cell no	1			2	3		4	
Sequencing run order	1st	2nd	3rd	3rd	1st	2nd	1st	2nd
Samples	SAS (PCR amplicons)	SAS (Native)	H103 (PCR amplicons)	H103 (Native)	SAS-R (Native)	SAS-R (PCR amplicons)	H103-R (Native)	H103-R (PCR amplicons)
Quality score > 5	18 909 (74.2%)	3819 (52.3%)	1866 (52.9%)	2774 (55.8%)	208 176 (99.9%)	25 820 (99.7%)	378 042 (99.9%)	53 200 (55.1%)
Quality score > 10	2275 (8.9%)	1951 (26.7%)	6 (17%)	516 (10.4%)	149045 (71.5%)	11 140 (43%)	287 765 (76.1%)	445 (0.5%)

### Distinct mtDNA mutational and methylation profiles in OSCC cells with acquired resistance to cisplatin

The variant analysis identified a total of 72 mutations in the samples (S3 Table). A majority of the mutations (*n* = 60) were synonymous single-base substitutions. So, their functional relevance was expected to be low. Ten mutations were missense base substitutions. Only two mutations were insertions. The variants predicted to be functionally significant are listed in Table 4. We further compared these predicted functional variants for their significance differences in the variant allele fractions between the cisplatin-resistant and parental cells with Bonferrani corrected *p*-value. From the analysis, the variants observed in the cisplatin-resistant cells were also present in the parental cells for both SAS and H103 cells, though variation in terms of the allele fractions of several variants was significantly observed between SAS and SAS-R cells.

**Table 4:** List of mtDNA variants discovered in SAS, H103, and cisplatin-resistant cells

MtDNA region	Mutation	Variant allele fraction				UniProtID; Amino acid position & alteration	Functional effects of variant score (Prediction)					
		SAS	SAS-R	H103	H103-R		PolyPhen-2 - HumDiv (Index)	PANTHER (Index)	Envision (Index)	Mutation Assessor (Index)	MutPred2 (Index)	SNPs&GO (Index)
*MT-ND1*	m.3910G > C	0.02^a^	0.23^b^			P03886; 202 E > Q	1.000 (ProbD)	4200 (ProbD)	0.87 (WT)	4.94 (High)	0.24 (Benign)	0.40 (Neutral)
*MT-ND2*	m.4833A > G	0.42	0.37			P03891; 122 T > A	0.665 (PossD)	NA	0.99 (WT)	1.03 (Low)	0.19 (Benign)	0.23 (Neutral)
*MT-ATP6*	m.8701A > G	0.63	0.64^b^			P00846; 59 T > A	0.002 (ProbB)	30 (ProbB)	0.93 (WT)	0.62 (Neutral)	0.08 (Benign)	0.22 (Neutral)
*MT-ATP6*	m.8860A > G	0.58	0.62^b^	0.68^a^	0.72	P00846; 112 T > A	0.000 (ProbB)	910 (ProbD)	0.93 (WT)	2.21 (Medium)	0.29 (Benign)	0.55 (Disease)
*MT-ND3*	m.10398A > G	0.58	0.71^b^			P03897; 114 T > A	0.000 (ProbB)	30 (ProbB)	0.98 (WT)	− 0.60 (Neutral)	0.2 (Benign)	0.03 (Neutral)
*MT-ND5*	m.13145G > A			0.38^a^	0.62	P03915; 270 S > N	0.000 (ProbB)	30 (ProbB)	0.95 (WT)	0.61 (Neutral)	0.04 (Benign)	0.16 (Neutral)
*MT-ND5*	m.13247 T > C			0.31^a^	0.38^a^	P03915; 304 F > S	0.997 (ProbD)	910 (ProbD)	0.90 (WT)	2.99 (Medium)	0.84 (Disease)	0.715 (Disease)
*MT-CYB*	m.14766C > T	0.69	0.66^b^	0.64^a^	0.70	P00156; 7 T > I	0.000 (ProbB)	30 (ProbB)	0.95 (WT)	2.62 (Medium)	0.06 (Benign)	NA
*MT-CYB*	m.14798 T > C	0.51	0.53^b^			P00156; 18 F > L	0.000 (ProbB)	324 (PossD)	0.90 (WT)	− 1.28 (Neutral)	0.13 (Benign)	0.18 (Neutral)
*MT-CYB*	m.15326A > G	0.76	0.66^b^	0.70	0.67	P00156; 194 T > A	0.000 (ProbB)	91 (ProbB)	0.95 (WT)	− 1.07 (Neutral)	0.09 (Benign)	0.19 (Neutral)

^a^Variant allele fraction was calculated from the base statistics from Integrative Genomics Viewer version 2.3.97, where the minimum allele coverage was set to nine and the minimum number of variant reads was set to three

^b^Fisher’s exact test for differences in the variant allele fractions between SAS and SAS-R cells. Bonferonni corrected *p*-value; *p* < 0.00625

PolyPhen-2 index: Possibility damaging (ProbD), probability damaging (ProbB), and probably benign (ProbB) indicate substitution is predicted to be damaging with high confidence, to be benign with high confidence, and to be damaging but with low confidence, respectively

PANTHER index: The conservation of mutation is calculated based on the preservation time in million years, whereby the preservation time < 200, between 200 and 450, and > 450 million years indicate the prediction of probably damaging (ProbD), possibly damaging (PossD), and probably benign (ProbB) mutation, respectively

Envision index: The prediction score of the mutational effect ranging from ~ 0 (most damaging) to 1 (most wild-type-like/WT)

MutationAssessor index: High and medium indicate the mutation is likely functional; Low and neutral indicate the mutation is likely non-functional

MutPred2 index: The pathogenicity score of the mutation ranging from 0 – 1 (Benign to Disease)

SNPs&GO index: A mutation with the probability score > 0.5 is predicted to be a disease-related polymorphism (Disease), while score < 0.5 is predicted to be a neutral-related polymorphism (Neutral)

The variant allele fraction from each native sample was computed based on the fraction of the base-called reads that supported the variants from Nanopolish, or the base statistics from Integrative Genomics Viewer version 2.3.97. The functional effects of the variants were predicted from a variety of open-source algorithms. A variant was classified as functional when it was predicted by at least three algorithms to be damaging

Importantly, the variant analysis significantly detected a m.3910G > C mutation in the *MT-ND1* gene of SAS-R cells. The mutation occurred in the parental SAS cells at a very low allele fraction (Table 4; *p* = 0.0032). We inspected the sequence alignment in Integrative Genomics Viewer to confirm the presence of the mutation, which involved an amino acid change of glutamic acid to glutamine in NADH dehydrogenase subunit 1 of mitochondrial complex I. The significant enrichment of the mutation, along with the predictions by different algorithms that it could be functionally damaging (Table 4), suggested potential involvement in the development of cisplatin resistance. Meanwhile, no significant difference in allele fractions of mtDNA variants was observed between H103 and H103-R cells.

An analysis of methylation status revealed that CpG methylation was present across the mitochondrial genomes of all four cell lines, albeit at low levels of about 3–7% (Table 5). Interestingly, both SAS-R and H103-R cells exhibited changes in methylation patterns. Three CpG sites, located in the mitochondrial light-strand promoter (LSP) region of the displacement loop (D-loop) and the gene bodies encoding *MT-ND4* and *MT-ND5*, were hypermethylated in SAS-R cells when compared to the parental SAS cells (Table 6). H103-R cells exhibited four hypermethylated CpG sites relative to the parental H103 cells. The CpG sites are located within the gene bodies of mitochondrial ribosomal RNA 16S (*MT-RNR2*), *COX1* (*MT-CO1*), and *CYTB* (*MT-CYB*), which encode components of mitochondrial specific ribosome, complex IV and complex III, respectively (Table 7).

**Table 5:** The level of CpG methylation in the mitochondrial genomes of all the experimental cell samples

Sample	Total called CpG sites	Methylated CpG sites	Level of CpG methylation (%)
SAS	14077	888	6.31
SAS-R	189846	6049	3.19
H103	3094	186	6.01
H103-R	57947	1826	3.15

The CpG methylation level represents the percentage of methylated sites over total called sites (methylated and unmethylated CpG sites) across whole mitochondrial genome

**Table 6:** Differences in the methylation of the CpG sites in the mitochondrial genomes of SAS cells and their cisplatin-resistant cells, as analysed by MOABS

CpG site	Gene region	SAS		SAS-R		Credible methylation difference
		Total called sites	Methylated frequency	Total called sites	Methylated frequency	
411	D-loop	174	0.24	17	0.77	0.30
11689	*MT-ND4*	1020	0.08	76	0.37	0.20
12456	*MT-ND5*	112	0.38	5	1	0.22

A CpG site was considered differentially methylated between two samples when the credible methylation difference exceeded 0.2

**Table 7:** Differences in the methylation of the CpG sites in the mitochondrial genomes of H103 cells and their cisplatin-resistant cells, as analysed by MOABS

CpG site	Gene region	H103		H103-R		Credible methylation difference
		Total called sites	Methylated frequency	Total called sites	Methylated frequency	
2332	*MT-RNR2*	130	0.03	9	0.56	0.26
5909	*MT-CO1*	489	0.04	21	0.43	0.23
7160	*MT-CO1*	276	0.08	14	0.57	0.28
15698	*MT-CYB*	77	0.14	6	0.83	0.32

A CpG site was considered differentially methylated between two samples when the credible methylation difference exceeded 0.2

### Altered mtDNA content in cisplatin-resistant OSCC

**Fig. 1:**
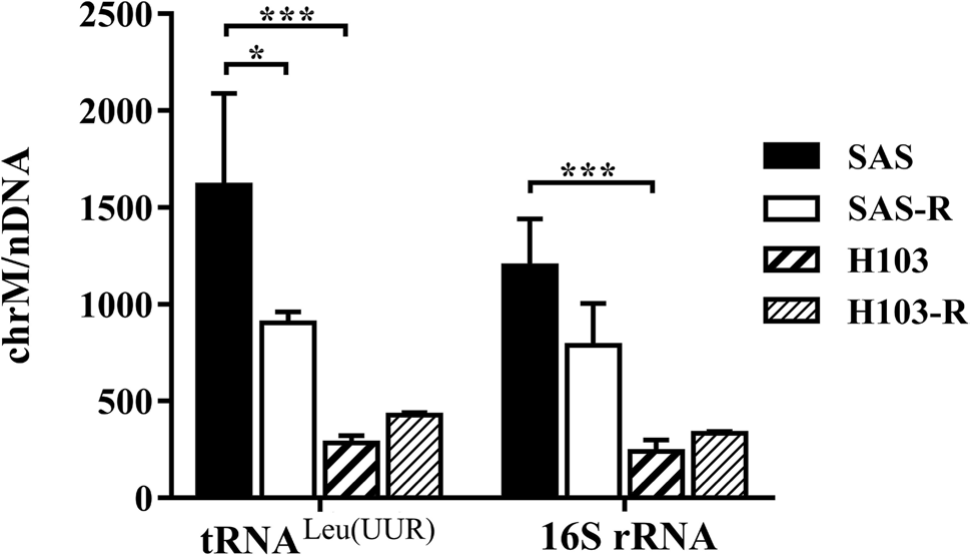
Estimation of mtDNA content via qPCR analysis. The amplification levels of two mitochondrial genes, tRNA^Leu(UUR)^ and 16S rRNA, were normalized against that of a nuclear gene, β2-microglobulin. Data are presented as mean ± SD. * *p* < 0.05, *** *p* < 0.001, *n* = 3. *chrM* mitochondrial chromosome, *nDNA* nuclear DNA

We found an interesting relation between mtDNA content and cisplatin responsiveness. SAS-R cells had significantly lower mtDNA content than the parental SAS cells (Fig. 1, tRNA^Leu(UUR)^, *p* = 0.0322). Similarly, both H103 (tRNA^Leu(UUR)^, *p* = 0.0004; 16S rRNA, *p* = 0.0002) and H103-R cells had less mtDNA than the more cisplatin-sensitive SAS cells. There was, however, no significant difference in mtDNA content between H103-R and H103 cells.

### Mitochondrial OCR was decreased in cisplatin-resistant OSCC cells

**Fig. 2:**
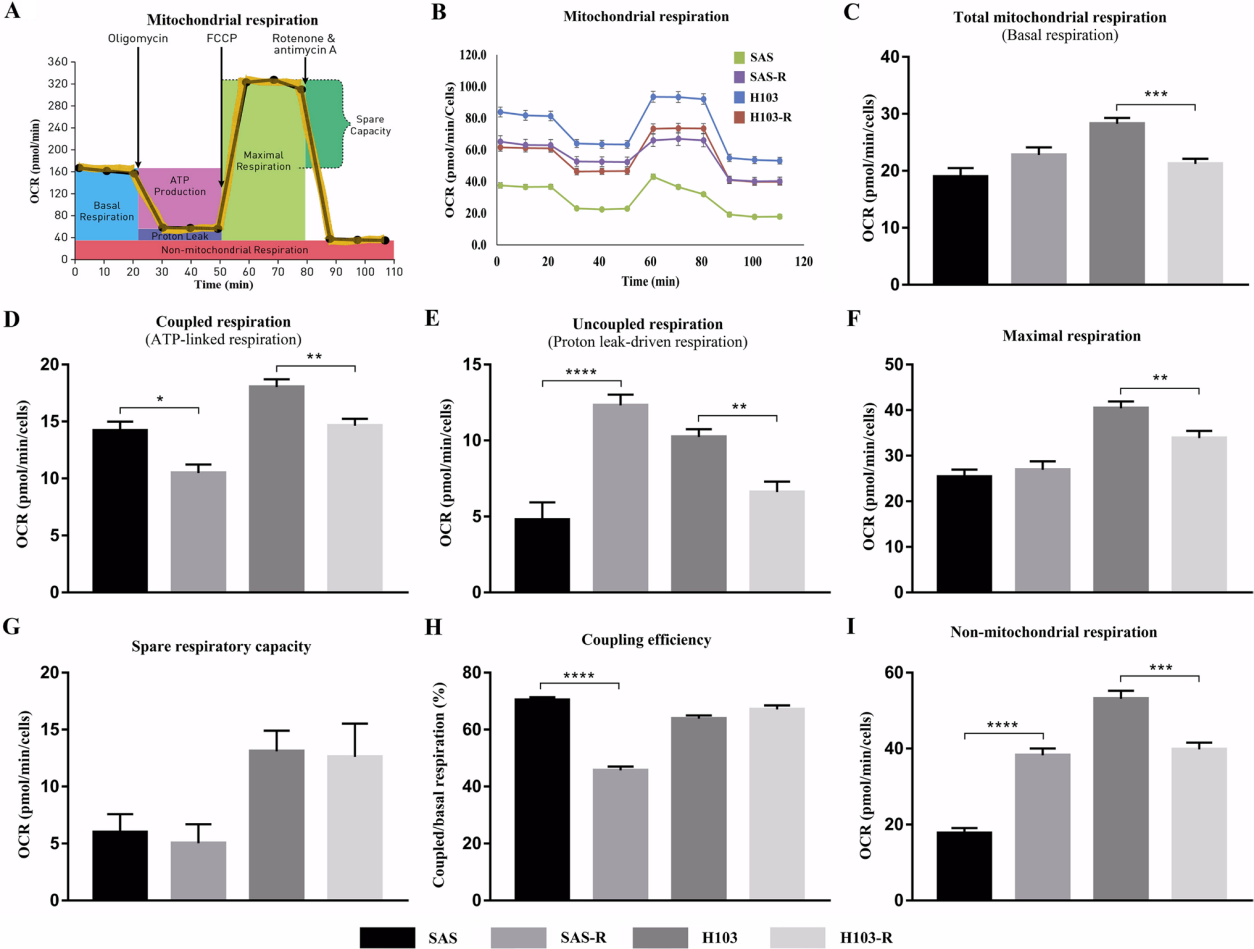
A summary of mitochondrial respiration profiles of the cisplatin-resistant cells and their parental SAS and H103 cells obtained via mitochondrial OCR analyses using Seahorse XF Cell Mito Stress Test kit. **A** Seahorse XF Cell Mito Stress Test profile of the key parameters of the mitochondrial respiration. **B** The representative OCR profiles of SAS, H103 and their cisplatin-resistant cells. **C** The total mitochondrial respiration or basal respiration was determined by total OCR that responsible for both (**D**) coupled and (**E**) uncoupled respiration. **G** The spare respiratory capacity was determined by the difference between (**F**) maximal and (**C**) basal respiration. **H** The coupling efficiency, referred to the efficiency of the mitochondria to utilize the oxygen and respiration substrate to generate ATP, was presented as percentage of the ATP-linked respiration to the total mitochondrial respiration. The remaining OCR was presented as (**I**) the OCR that was not related to mitochondrial respiration. The data are presented in means ± SD. * *p* < 0.05, ** *p* < 0.01, *** *p* < 0.001, **** *p* < 0.0001, against their respective parental cells, *n* = 3

The mitochondrial respiratory functions of SAS, H103, and the cisplatin-resistant cells were evaluated by measurements of the OCRs during oxidative phosphorylation (OXPHOS). In brief, the cellular oxygen concentrations were sequentially measured over time to provide the total cellular OCR profiles for direct correlation to key mitochondrial respiratory parameters, namely basal and maximal respiration, adenosine triphosphate (ATP) production, proton leak, coupling efficiency, and spare respiratory capacity, as depicted in Fig. 2 (Fig. 2A).

In the comparison of SAS and SAS-R cells, the mitochondrial respiration was notably compromised in the latter, as evidenced by a reduction in ATP-link respiration (Fig. 2D) despite an increase in the total mitochondrial respiration (Fig. 2C). The elevated total mitochondrial respiration was largely driven by proton leaks (Fig. 2E) rather than coupled respiration, the normal mode of ATP synthesis (Fig. 2D). The other observations that indicated impaired mitochondrial respiratory function in SAS-R cells were decreased mitochondrial coupling efficiency (Fig. 2H) and increased uncoupled and non-mitochondrial respiration (Fig. 2E, I, respectively), along with unchanged spare respiratory capacity (Fig. 2G). In contrast, H103-R cells showed no difference in coupling efficiency compared to the parental H103 cells (Fig. 2H); but the total mitochondrial respiration (Fig. 2C) was reduced by decreases in both coupled and uncoupled respiration (Fig. 2D, E). This suggests a general reduction in cellular bioenergetics rather than abnormalities in the mitochondrial respiration per se.

### OSCC subtypes showed distinct ΔΨm profiles

**Fig. 3:**
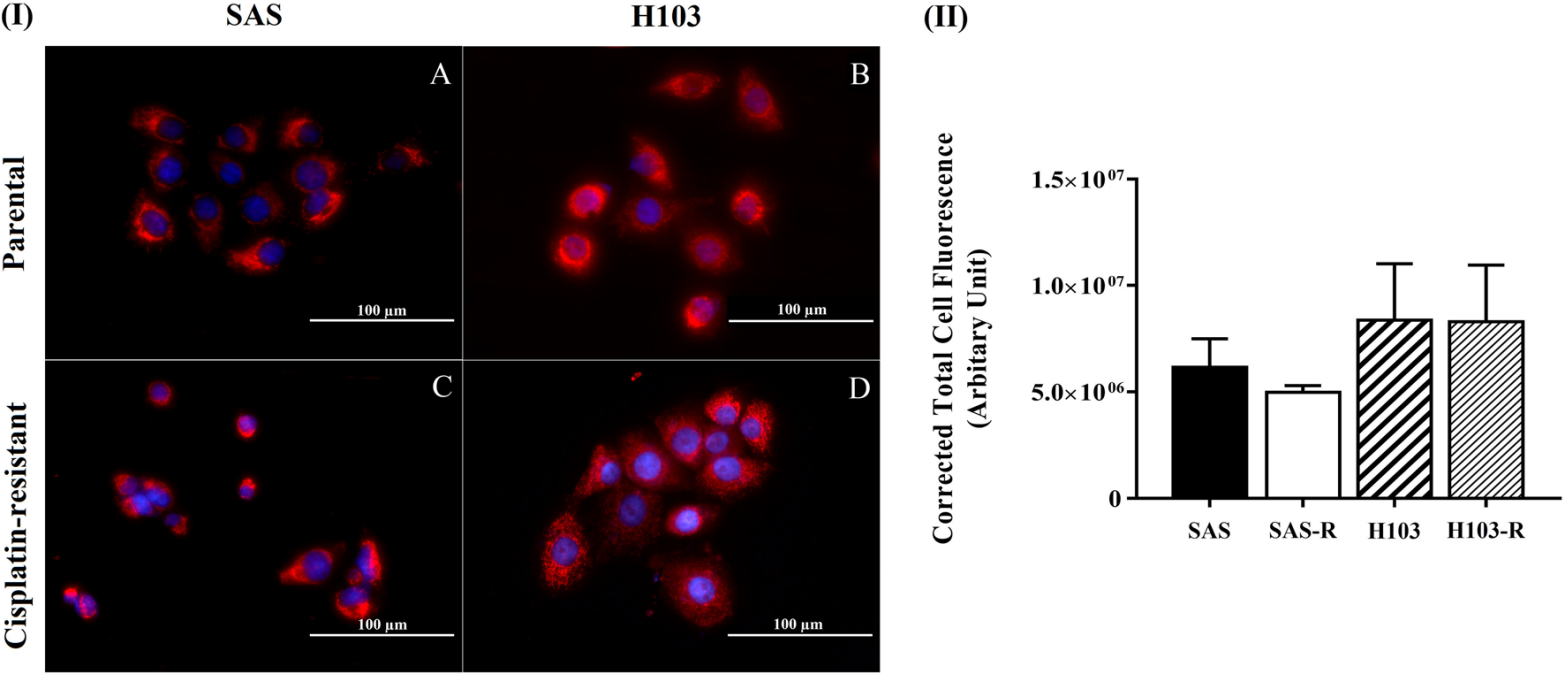
Mitochondrial membrane potential of SAS, H103 and their parental cells as evaluated via MitoTracker Red CMXRos staining. (I) Representative fluorescence images of (**A**) SAS, (**B**) H103, (**C**) SAS-R, and (**D**) H103-R cells at 400 × magnification. Red and blue colours represent mitochondria and nucleus staining respectively. (II) Quantification of the fluorescence intensities of the mitochondria staining using background-subtracted intensities of the selected area of the cells. The intensities of 30 individual cells were measured per experimental group. Data are presented as mean ± SD. *n* = 3

Altered ΔΨm is one of the key indicators of defective mitochondrial respiratory function [33]. In this study, the differences in ΔΨm between SAS-R and H103-R cells and the parental cells were determined by MitoTracker Red CMXRos staining. The accumulation of the dyes in the mitochondria depends on ΔΨm, which can be either hyperpolarized or hypopolarized [34]. From the findings, SAS-R cells had lower ΔΨm and were thus less intensely stained than the parental SAS cells (Fig. 3; *p* > 0.05). No difference in ΔΨm was observed between H103-R and H103 cells, supporting the findings on the mitochondrial coupling efficiency from the OCR analyses.

### Cisplatin-resistant OSCC cells exhibited enhanced ability to evade cisplatin-induced oxidative stress

**Fig. 4:**
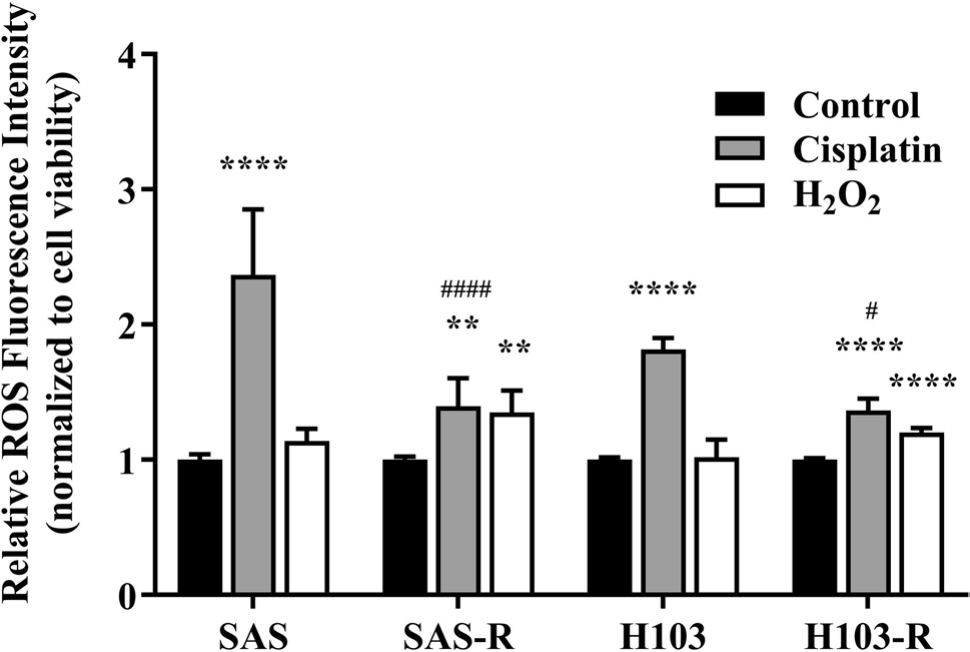
Measurement of the changes in intracellular ROS levels after treatment with cisplatin for 72 h. The data are presented in means ± SD of ROS levels relative to the untreated control group and normalized against the percentage of viable cells. ** *p* < 0.01, **** *p* < 0.0001, against respective untreated control groups in each cell groups. ^#^ *p* < 0.05, ^####^ *p* < 0.0001, against respective parental cell groups. *n* = 3

One of the mechanisms of action of cisplatin is the induction of ROS production to trigger oxidative stress and activate DNA damage responses that eventually culminate in cell death. Intracellular ROS is mostly produced in mitochondria, specifically at mitochondrial complexes I, II, and III [35–38]. In this study, the intracellular ROS level was measured to determine the extent of the oxidative stress induced by cisplatin. Both SAS-R and H103-R cells were noticeably more resistant to the ROS-inducing action of cisplatin than the parental cells (Fig. 4).

## Discussion

Accumulation of abnormalities in the mitochondrial genome is common during the development and progression of cancer [39, 40]. Decreased susceptibility to chemotherapy-induced cytotoxicity is one of the adaptive mechanisms that give cancer cells survival advantages [15, 41, 42]. In this work, we derived cisplatin-resistant cells from the SAS and H103 cell lines via continual treatments with cisplatin, according to a published protocol [11]. Then we sequenced the mitochondrial genomes and measured the mitochondrial respiratory function of the cells and their capacity to generate intracellular ROS to investigate the molecular mechanisms underlying drug resistance in OSCC. As mtDNA encodes essential components of the OXPHOS system, mtDNA alterations may affect mitochondrial respiration and the production of ROS.

Overall, we demonstrated that prolonged cisplatin treatment for about 4 months induced drug resistance, yielding IC_50_ 1.5–2 times higher than the pre-treatment values, consistent with the levels of therapeutic resistance observed clinically [11, 43]. In both SAS and H103 cells, underlying changes in mtDNA were probably culpable of the diminished drug sensitivity. Cells harbouring survival-enhancing mtDNA mutations gained dominance; methylation patterns were altered to regulate gene transcription; and mtDNA content was curtailed and maintained just above the tumorigenic threshold. Furthermore, we showed that SAS-R and H103-R cells, consistent with their reduced mitochondrial respiratory function, as indicated by reduced coupled respiration and/or ΔΨm, were less sensitive to ROS attack by cisplatin. Our findings suggest that mtDNA alterations may modulate cisplatin sensitivity by modifying mitochondrial functions, possibly through altering the structure, function, or expression of the proteins involved in the mitochondrial respiration or ROS production. Accumulating evidence has supported the possible links between mtDNA alterations, defective mitochondrial functions, apoptosis, and drug resistance. Reduced mitochondrial respiratory function is often accompanied by reduced ΔΨm, especially when the impaired activity is due to mutations in the protein complexes that make up the electron transport chain, specifically complex I, III, and IV [44]. A prior study using cytoplasmic hybrid cell lines has reported that mitochondrial ROS response is crucial for cisplatin cytotoxicity, and consequently, cells lacking mtDNA and functionally intact mitochondria can become cisplatin-resistant [45]. Previous studies on hepatoma and small cell lung cancer reported that low mtDNA content reduced the sensitivity of cancer cells to ROS-induced cytotoxicity by impairing mitochondrial respiration, depolarizing the inner mitochondrial membrane potential, and causing a compensatory increase in the expression of antioxidant enzymes [46, 47].

Most notably, an originally undetected *MT-ND1* mutation in the SAS cells was significantly enriched, surpassing the variant-calling threshold. SAS and H103 are known to exhibit different clinicopathological characteristics, leading them to adopt unique self-defence mechanisms for survival and to respond differently to cisplatin [48, 49]. It is likely that the two cell lines are also genetically distinct, explaining why the *MT-ND1* mutation was detected only in SAS-R cells. We suggest that the emergence of the *MT-ND1* mutation in SAS cells may reflect a cisplatin-induced shift of the heteroplasmic (or polyploidy) mtDNA population in the malignant evolution of OSCC. The co-existence of wild-type and mutant mtDNA masks the pathogenicity of heteroplasmic mutations, permitting penetrance only when a certain threshold of mutation load, heteroplasmy level, is exceeded [50, 51]. Heteroplasmy levels are tissue-specific [52] and could be influenced by several mechanisms, including random segregation during cellular division, dynamic mitochondrial fusion and fission, mtDNA replication rates, and intercellular mitochondrial transfer during clonal expansion [53]. Understanding the functional significance of induced heteroplasmycan help uncover fundamental aspects of tumour survival and resistance. Exploiting this dynamic intercellular mitochondrial communication could lead to more targeted cancer treatments, such as mitochondrial transplantation therapy, which involves introducing healthy mitochondria or mtDNA into cancer cells [54–56].

Further studies are required to validate the functional impact of the enriched low-frequency *MT-ND1* mutation, such as screening of additional OSCC cell lines or clinical samples. Detecting the mutation in a larger sample group may confirm its subtype-specific role in modulating cisplatin sensitivity, providing a basis for genetics-guided and better-informed OSCC treatments. An important implication of such variable and subtype-specific mtDNA profiles is that, in predicting cisplatin sensitivity, a sequencing-based method would be more suitable than a targeted assay that detects only a limited number of known mtDNA mutations. The small size of the mitochondrial genome means that mtDNA sequencing is technically less challenging than a typical high-throughput multi-gene panel.

We further demonstrated that SAS-R cells had less mtDNA, supporting our previous hypothesis that low mtDNA content was associated with cisplatin resistance in OSCC [15]. Other studies have shown that cells adjust mtDNA content to meet their energy demand under different conditions, such as oxidative stress and cancer [57, 58]. For example, in response to increased stress caused by mitochondrial dysfunction or adverse environmental changes, mitochondria send mitostress signals to the nucleus. Then, the nucleus responds by activating stress response signalling pathways that redirect cellular metabolism to sustain bioenergetics [4, 59]. Glycolysis is commonly augmented at the expense of mitochondrial activity, resulting in low mtDNA content [58, 60]. This interplay between the nucleus and mitochondria in metabolic reprogramming enables cells to adapt energy production mechanisms for survival under stress conditions. Additionally, mtDNA replication depends on the nuclear-encoded protein machinery. For instance, cells with mutations in mitochondrial transcription factor A (TFAM), a key nuclear-encoded protein that regulates mtDNA replication and transcription, exhibited reduced mtDNA content, mitochondrial dysfunction, and decreased sensitivity to cisplatin treatment [45].

However, no significant difference in mtDNA content was found between H103 and H103-R cells. This may be explained by the originally low mtDNA content of H103 cells, which prevented it from being reduced below the cell-specific threshold required for tumourigenesis. It has been reported that mtDNA depletion decreased or even eradicated tumourigenicity [61]. Hence, cancer cells carefully restrict mtDNA replication to maintain a minimum mtDNA level necessary for sustaining tumourigenicity. The attendant decline in mitochondrial activity shifts the usual mode of ATP synthesis towards glycolysis in the maintenance of cell proliferation [62]. In this study, we demonstrated that such preference for glycolytic metabolism decreased the mitochondrial respiratory function in H103-R cells considerably, even though the inherent ability of mitochondria to perform cellular respiration was largely unaffected.

In our previous work, we identified a link between mtDNA methylation within gene bodies and cisplatin responsiveness in OSCC, where three hypermethylated CpG sites within the *MT-CO1* and *MT-CYB* genes were thought to reduce cisplatin sensitivity in H103 cells, and the expression levels of the encoded genes were higher, albeit marginally, than SAS cells [15]. In the current study, we also detected changes in mtDNA methylation within the gene bodies after repeated cisplatin treatments of both SAS and H103 cells. This suggests that gene body methylation could govern the development of drug resistance, presumably by regulating mtDNA transcription. However, this is still a subject of debate with conflicting findings [63, 64].

Earlier evidence has also suggested that methylation within the mitochondrial D-loop could regulate the replication and transcription of the mitochondrial genome [65, 66]. In general, mitochondrial D-loop acts as the control region of mtDNA, where polycistronic transcription is initiated at the heavy-strand promoter (HSP) and LSP. Methylation at these promoter sites affects the binding of mitochondrial nucleoid, a histone-like structure, to mtDNA and prevents the recruitment of other transcription factors and consequently transcription initiation [67]. Importantly, the mechanism that drives mtDNA replication is linked to that of mtDNA transcription. A short mitochondrial RNA transcript from LSP serves as the primer for the replication of nascent H-strand [7, 68–70]. Coincidentally, we detected a hypermethylated CpG site in the LSP region of the SAS-R cells, and this may explain their lower mtDNA content than the SAS cells.

Several limitations of our study warrant consideration. The lack of in-depth validation by mechanistic experiments is a major limitation of this study. Specifically, the direct association between the mtDNA alterations, mitochondrial dysfunction, and cisplatin resistance was not confirmed. Using gene editing to reverse mutations predicted to promote cancer progression, particularly the *MT-ND1* mutation, and ascertaining the effect on cisplatin sensitivity can be an effective method for confirming the functional significance of the mtDNA variants. Additionally, a mouse xenograft model of OSCC can be used to further evaluate the functional effects of the mtDNA variant on cisplatin resistance in vivo. Similar strategies, such as mtDNA elimination and DNA demethylation, can be used to validate the association of mtDNA content and methylation status with therapeutic resistance in OSCC. Lastly, we did not sequence the nuclear genomes, so the contribution of genomic mutations to the development of cisplatin resistance could not be determined. Nevertheless, this study revealed how quickly resistance-inducing mtDNA alterations could emerge and potentially diminish cisplatin responsiveness.

## Conclusion

Overall, this study highlights the potential role of heteroplasmic mtDNA mutations in modulating the adaptation of specific OSCC subtypes to cisplatin treatments by characterizing both mitochondrial genome and functions of resistant OSCC cells. These preliminary findings warrant further in vitro, in vivo, and clinical investigations to elucidate the mechanisms that underlie cisplatin resistance in OSCC; these mechanisms are likely co-mediated by both the nuclear and mitochondrial genomes. Firstly, whole-genome sequencing and transcriptomic profiling of OSCC could confirm the role of nuclear DNA in controlling mtDNA alterations. Secondly, DNA methylation editing, combined with relevant follow-up analyses, may clarify the influence of gene body methylation on mtDNA transcription. These additional insights could be valuable for the development of effective targeted therapies to eradicate therapeutic failure and relapses in patients with OSCC. The identified mtDNA alterations could be used as predictive genetic signatures to define OSCC patients most likely to benefit from the cisplatin chemotherapy and thus assist clinician to make better informed treatment decisions.

## Supplementary Information

Additional file 1: S1 Table: Details of MinION sequencing runs

Additional file 2: S2 Table: Primers used for mtDNA gene-specific qPCR

Additional file 3: S3 Table: List of mtDNA variants and their variant allele fraction in all cell samples

Additional file 4: S1 Appendix: A schematic overview of the development of cisplatin-resistant cells derived from the SAS and H103 cell lines

Additional file 5: S2 Appendix: Depth of sequencing coverage of DNA library of SAS-R and H103-R cells

Additional file 6: S1 Dataset: Raw data of cell viability percentage for cisplatin chemosensitivity study

Additional file 7: S2 Dataset: Raw data of the Cq values of three independent mtDNA gene-specific qPCR reactions and amplification efficiency of primers

Additional file 8: S3 Dataset: Raw data of the normalized oxygen consumption rate of three biological samples

Additional file 9: S4 Dataset: Raw data of the corrected total cell fluorescence of three independent measurement of mitochondrial membrane potential

Additional file 10: S5 Dataset: Raw data of intracellular oxygen reactive species measurement

## Data Availability

The raw nanopore sequencing datasets generated for this study can be found in the Sequence Read Archive (SRA; Accession No. PRJNA712949 and Accession No. PRJNA972870). The raw data of the other analyses presented in this study are provided as supplementary datasets (S1 Dataset–S5 Dataset).
