# Supplementary material for: Potential role of heteroplasmic mitochondrial DNA mutations in modulating the subtype-specific adaptation of oral squamous cell carcinoma to cisplatin therapy
Source: Discov Oncol. 2024 Oct 19;15:573. doi: 10.1007/s12672-024-01445-8 (PMC11490477; doi:10.1007/s12672-024-01445-8)
Supplement: Supplementary file 1 — Additional file 1: S1 Table: Details of MinION sequencing runs [file 12672_2024_1445_MOESM1_ESM.pdf]

Supplementary Information

S1 Table: Details of MinION sequencing runs.

Flow Cell (Sequencing chemistry)	Sequencing run order	Experimental cell sample	Input DNA	Sample processing
1 (1D)	First	SAS	PCR amplicons	Long PCR-amplification and purification
	Second	SAS	Native DNA	Linearization and purification
	Third	H103	PCR amplicons	Long PCR-amplification, purification, and limited barcoding PCR
2 (ID)	Third	H103	Native DNA	Linearization and purification
3 ($1D^2$)	First	SAS-R	Native DNA	Linearization and purification
	Second	SAS-R	PCR amplicons	Long PCR-amplification and purification
4 ($1D^2$)	First	H103-R	Native DNA	Linearization and purification
	Second	H103-R	PCR amplicons	Long PCR-amplification and purification
