# Supplementary material for: Potential role of heteroplasmic mitochondrial DNA mutations in modulating the subtype-specific adaptation of oral squamous cell carcinoma to cisplatin therapy
Source: Discov Oncol. 2024 Oct 19;15:573. doi: 10.1007/s12672-024-01445-8 (PMC11490477; doi:10.1007/s12672-024-01445-8)
Supplement: Supplementary file 2 — Additional file 2: S2 Table: Primers used for mtDNA gene-specific qPCR [file 12672_2024_1445_MOESM2_ESM.pdf]

Supplementary Information

S2 Table: Primers used for mtDNA gene-specific qPCR.

Gene	Description	Product length (bp)	Efficiency (%)	Primer sequences (5' to 3')	
MtDNA tRNA$^{Leu(UUR)}$	Microsomal glutathione S-transferase 1	107	101	Forward	CACCCAAGAACAGGGTTTGT
				Reverse	TGGCCATGGGTATGTTGTTA
B2M	Nuclear DNA β2-microglobulin	86	91	Forward	TGCTGTCTCCATGTTTGATGTATCT
				Reverse	TCTCTGCTCCCCACCTCTAAGT
MtDNA 16S rRNA	Serine palmitoyltransferase small subunit B	97	105	Forward	GCCTTCCCCCGTAAATGATA
				Reverse	TTATGCGATTACCGGGCTCT
