# Supplementary material for: Potential role of heteroplasmic mitochondrial DNA mutations in modulating the subtype-specific adaptation of oral squamous cell carcinoma to cisplatin therapy
Source: Discov Oncol. 2024 Oct 19;15:573. doi: 10.1007/s12672-024-01445-8 (PMC11490477; doi:10.1007/s12672-024-01445-8)
Supplement: Supplementary file 3 — Additional file 3: S3 Table: List of mtDNA variants and their variant allele fraction in all cell samples [file 12672_2024_1445_MOESM3_ESM.pdf]

Supplementary Information

S3 Table: List of mtDNA variants and their variant allele fraction in all cell samples.

MtDNA region	Mutation	Variant allele fraction					
		**SAS**	**SAS-R**	***p*-value**	**H103**	**H103-R**	***p*-value**
D-loop	m.73A>G	0.22	0.64	0.1288	0.55[a]	0.62	0.162
D-loop	m.150C>T				0.52	0.79	0.3645
D-loop	m.260G>A	0.30[a]	0.58	0.2183			
D-loop	m.263A>G	0.58	0.63	<0.0001	0.57[a]	0.64	0.1126
D-loop	m.282T>C				0.45	0.42	0.1432
D-loop	m.309insC	0.10[a]	0.06[a]	0.1936			
D-loop	m.315insC				0.40[a]	0.65[a]	>0.9999
D-loop	m.489T>C	0.41	0.65	0.7594			
MT-RNR1	m.709G>A	0.75	0.76	<0.0001			
MT-RNR1	m.750A>G	0.64	0.56	<0.0001	0.79[a]	0.65	0.0012
MT-RNR1	m.1438A>G	0.51	0.56	0.0026	0.53[a]	0.61	0.307
MT-RNR2	m.1811A>G				0.44[a]	0.59	0.6061
MT-RNR2	m.2706A>G	0.64	0.70	0.0002	0.73[a]	0.72	0.03
MT-ND1	m.3738C>T				0.91[a]	0.79	0.0033
MT-ND1	m.3910G>C	0.02[a]	0.23	0.0032			
MT-ND1	m.4107C>T	0.23[a]	0.26	0.7248			
MT-ND2	m.4505C>T	0.67	0.71	0.0007			
MT-ND2	m.4769A>G	0.37	0.65	0.8663	0.50[a]	0.65	0.5519
MT-ND2	m.4833A>G	0.42	0.37	0.0526			
MT-ND2	m.5108T>C	0.59	0.57	0.0106			
MT-ND2	m.5240A>G				0.54[a]	0.72	0.4056
MT-TA	m.5601C>T	0.34	0.50	>0.9999			
MT-CO1	m.6392T>C				0.17[a]	0.66	0.1362
MT-CO1	m.6455C>T				0.55[a]	0.54	0.2101
MT-CO1	m.6737A>G	0.62	0.80	0.0663			
MT-CO1	m.7028C>T	0.68	0.69	0.0074	0.46[a]	0.71	0.7791
MT-CO1	m.7055A>G				0.46[a]	0.45	0.3584
MT-CO2	m.7600G>A	0.63	0.64	<0.0001			
MT-ATP6	m.8701A>G	0.63	0.64	<0.0001			
MT-ATP6	m.8860A>G	0.58	0.62	0.0003	0.68[a]	0.72	0.0242
MT-ATP6	m.9165T>C	0.75	0.59	<0.0001			
MT-CO3	m.9365C>T				0.33[a]	0.66	>0.9999
MT-CO3	m.9377A>G	0.58	0.71	0.0064			
MT-CO3	m.9540T>C	0.69	0.72	<0.0001			
MT-CO3	m.9575G>A	0.62	0.66	<0.0001			
MT-CO3	m.9698T>C				0.67[a]	0.57	0.0835
MT-ND3	m.10398A>G	0.58	0.71	0.0029			
MT-ND3	m.10400C>T	0.63	0.69	<0.0001			

MT-ND4L	m.10733C>T				0.60[a]	0.71	0.3334
MT-ND4	m.10873T>C	0.26[a]	0.24	0.3585			
MT-ND4	m.11465T>C				0.55[a]	0.48	0.1899
MT-ND4	m.11467A>G				0.64[a]	0.59	0.1117
MT-ND4	m.11719G>A	0.73	0.75	<0.0001	0.67[a]	0.77	0.1895
MT-ND4	m.11809T>C	0.65	0.58	<0.0001			
MT-TL2	m.12308A>G				0.50[a]	0.53	0.3556
MT-TL2	m.12311T>C	0.58	0.53	<0.0001			
MT-ND5	m.12372G>A				0.30[a]	0.71	0.5344
MT-ND5	m.12705C>T	0.70	0.73	<0.0001			
MT-ND5	m.13145G>A				0.38[a]	0.62	>0.9999
MT-ND5	m.13247T>C				0.31[a]	0.38[a]	0.7605
MT-ND5	m.13563A>G	0.64	0.66	<0.0001			
MT-ND5	m.13677C>T	0.36	0.52	0.798			
MT-ND6	m.14200T>C	0.54	0.51	0.0002			
MT-ND6	m.14281C>T	0.31	0.28	0.07			
MT-ND6	m.14569G>A	0.59	0.66	0.0004			
MT-CYB	m.14766C>T	0.69	0.66	<0.0001	0.64[a]	0.70	0.1016
MT-CYB	m.14783T>C	0.59	0.64	0.0003			
MT-CYB	m.14798T>C	0.51	0.53	0.0021			
MT-CYB	m.15043G>A	0.67	0.74	<0.0001			
MT-CYB	m.15301G>A	0.42	0.48	0.0506			
MT-CYB	m.15326A>G	0.76	0.66	<0.0001	0.70	0.67	0.008
MT-TT	m.15924A>G	0.60	0.61	<0.0001			
D-loop	m.16146A>G				0.27[a]	0.55	0.4047
D-loop	m.16184C>A	0.21[a]	0.29[a]	>0.9999			
D-loop	m.16186C>T	0.13[a]	0.36[a]	0.007			
D-loop	m.16189T>C	0.15[a]	0.13[a]	0.5107			
D-loop	m.16223C>T	0.67	0.70	<0.0001			
D-loop	m.16260C>T				0.29[a]	0.48	0.8245
D-loop	m.16269A>G	0.64	0.59	<0.0001			
D-loop	m.16278C>T	0.74	0.77	<0.0001			
D-loop	m.16342T>C				0.37	0.75	0.6994
D-loop	m.16362T>C	0.44	0.40	0.003			

[a] Variant allele fraction was calculated from the base statistics from Integrative Genomics Viewer version 2.3.97, where the minimum allele coverage was set to nine and the minimum number of variant reads was set to three.
