# Supplementary material for: Potential role of heteroplasmic mitochondrial DNA mutations in modulating the subtype-specific adaptation of oral squamous cell carcinoma to cisplatin therapy
Source: Discov Oncol. 2024 Oct 19;15:573. doi: 10.1007/s12672-024-01445-8 (PMC11490477; doi:10.1007/s12672-024-01445-8)
Supplement: Supplementary file 4 — Additional file 4: S1 Appendix: A schematic overview of the development of cisplatin-resistant cells derived from the SAS and H103 cell lines [file 12672_2024_1445_MOESM4_ESM.pdf]

Supplementary Information

S1 Appendix: A schematic overview of the development of cisplatin-resistant cells derived from the SAS and H103 cell lines.

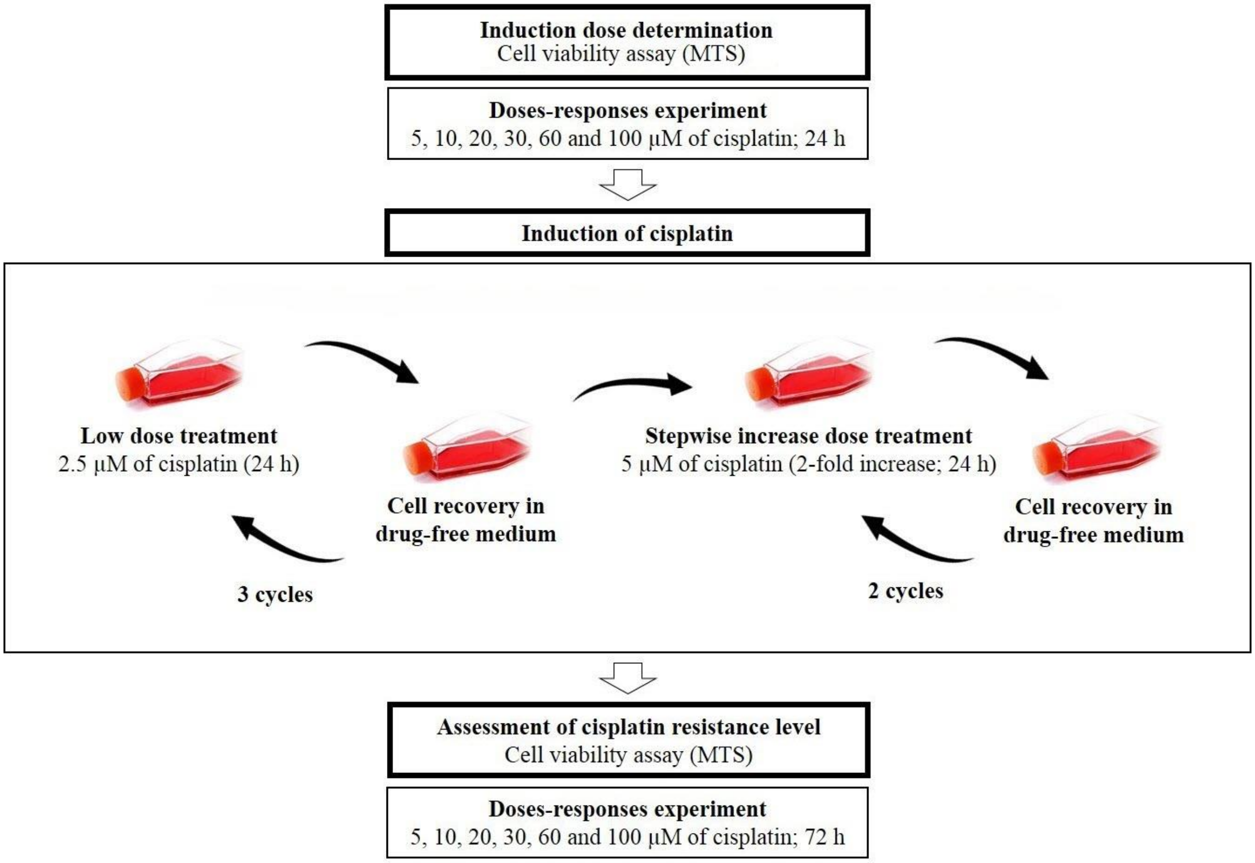

Fig. 1 A schematic overview of the development of cisplatin-resistant cells derived from the SAS and H103 cell lines. Initial dose-response experiments of cisplatin (5 − 100 µM) over 24 h were first performed and acute IC_{50} values of cisplatin for each cell line were evaluated to determine the dose for cisplatin induction. In general, cisplatin-resistant cells derived from SAS and H103 cells were established by repeated stepwise treatments with cisplatin. Cells were first seeded in a 25 cm^2 T-flask at a low cell density (20 – 30 % confluency). Drug treatments were begun on the next day and the first dose of cisplatin used was approximately 10 % and 5 % of the acute IC_{50} of cisplatin against SAS and H103 respectively. Lower initial dose of cisplatin was used for H103 as 10 % of its acute IC_{50} dose resulted in total loss of cell viability. After 24 h, the treatment was removed, and cells were allowed to recover in drug-free medium until cells become confluence. Upon confluence, cells can be subcultured, expanded and frozen down. The cells were frozen down for every single post-treatment to allow for maintenance of the cell stock in any case of loss of the evolving drug-resistant cells throughout the development. In subsequent passages, the cells were treated again using the same dose of cisplatin. The increment of dose was performed

at cautious based on time taken for the cells to recover and become confluence. Briefly, the cells were treated with 2.5 µM of cisplatin in the first three treatments followed by 5 µM of cisplatin for another two treatments for over four months. The 72-h IC_{50} doses were assessed and compared to that of their parental cells to confirm the increased level of drug resistance. The derived resistant cells were maintained in drug-free medium. The 72-h IC50 doses were re-assessed until four passages in drug-free medium to confirm no loss of resistance.
