# Supplementary material for: Potential role of heteroplasmic mitochondrial DNA mutations in modulating the subtype-specific adaptation of oral squamous cell carcinoma to cisplatin therapy
Source: Discov Oncol. 2024 Oct 19;15:573. doi: 10.1007/s12672-024-01445-8 (PMC11490477; doi:10.1007/s12672-024-01445-8)

Supplementary Information

S2 Appendix: Depth of sequencing coverage of DNA library of SAS-R and H103-R cells.

Fig 1: Depth of sequencing coverage of native DNA library of SAS-R cells.

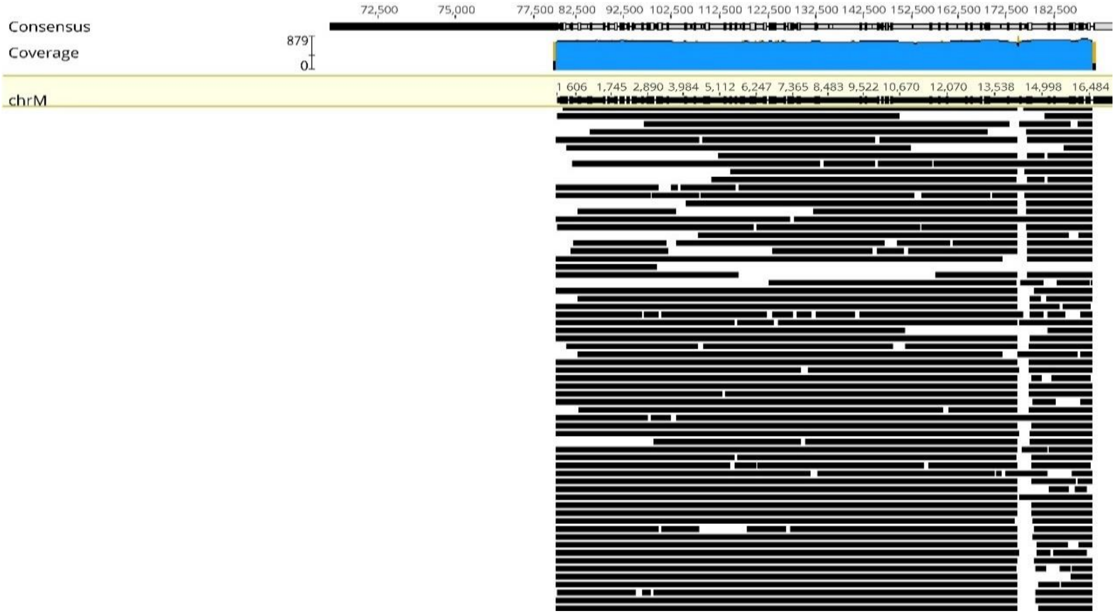

Fig 2: Depth of sequencing coverage of PCR amplicon library of SAS-R cells.

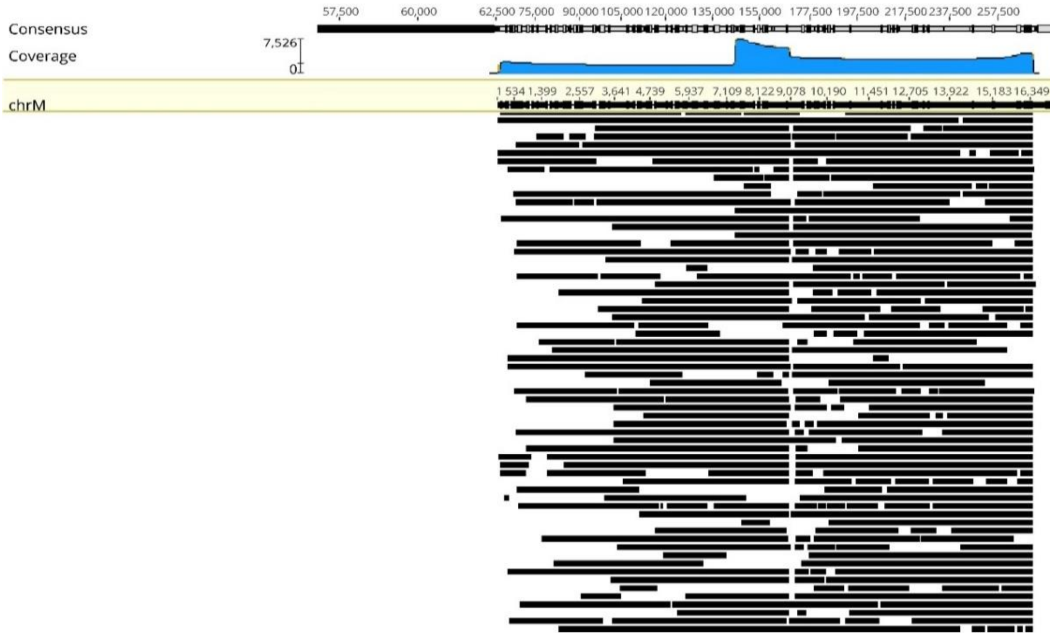

Fig 3: Depth of sequencing coverage of native DNA library of H103-R cells.

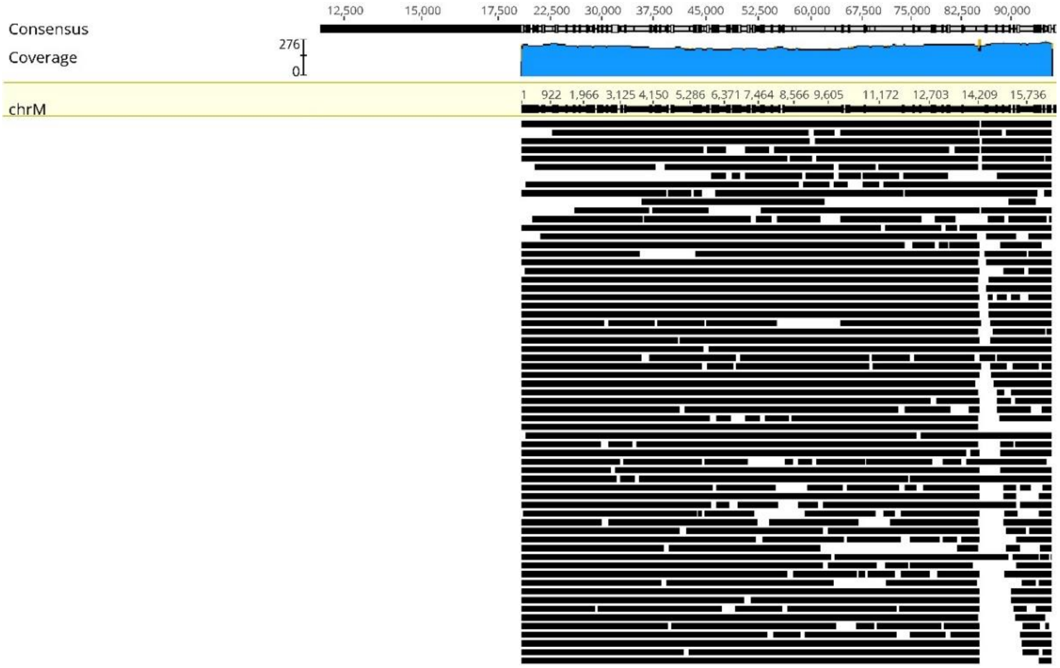

Fig 4: Depth of sequencing coverage of PCR amplicon library of H103-R cells.

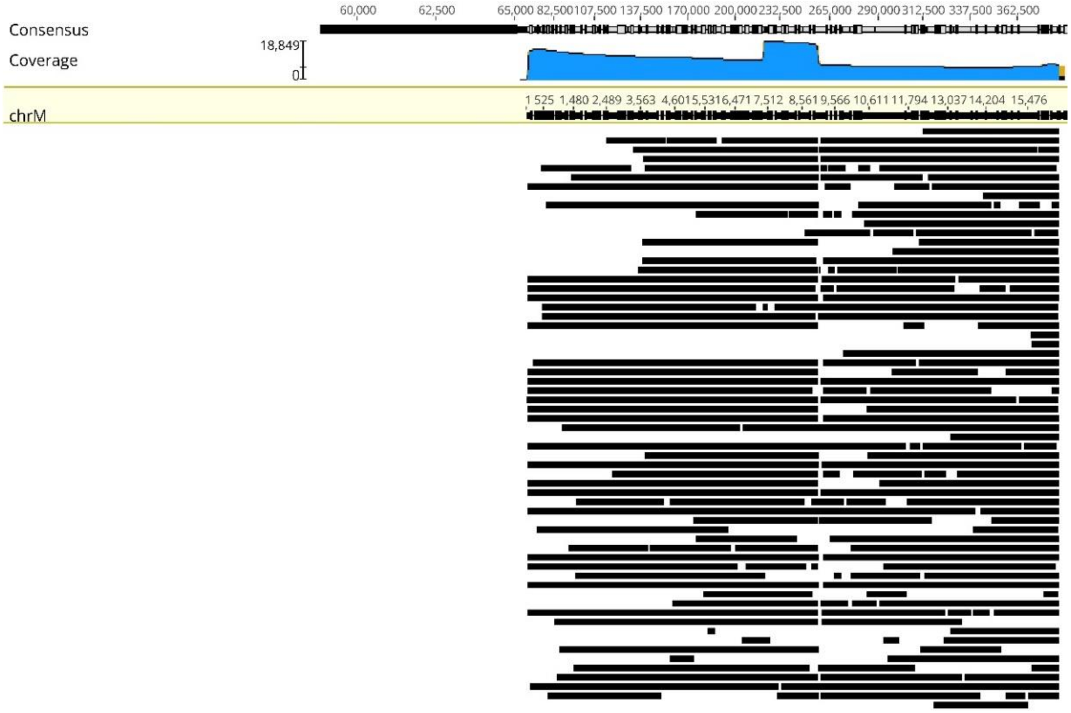

Supplement: Supplementary file 5 — Additional file 5: S2 Appendix: Depth of sequencing coverage of DNA library of SAS-R and H103-R cells [file 12672_2024_1445_MOESM5_ESM.pdf]
